# A Case of Lithotomy by the High Operation; with Remarks

**Published:** 1834-01-01

**Authors:** C. A. Key

**Affiliations:** Surgeon to Guy's Hospital.


*A Case of Lithotomy by the High Operation; with Remarks.* By C. A. KEY, Esq., Surgeon to Guy's Hospital.*

THIS case is interesting, from the circumstance of the high operation not having been frequently performed in this country, and from the cases in which it has been done not having been generally successful. During the last twenty years an operation of this kind has not been attempted in Guy's Hospital: in the male subject the lateral operation has been so successful, that no motive existed for changing the mode of operating; and, in the female, dilatation of the meatus has been resorted to with such satisfactory results, that the knife is but rarely employed. The following case is invested with an additional degree of interest, in consequence of the danger attending the operation being increased by the attack of scarlatina, on the fourth day after its performance.

Ann Gibbons, æt. eight and a half years, was admitted into Guy's Hospital, June 26th, 1833, under the care of Mr. Key. She complained of symptoms of stone in the bladder, viz. sharp cutting pains before making water, which she was obliged to do every three or four hours; after which the pain continued for some minutes, and then ceased. These symptoms first made their appearance when she was about four

* The case is drawn up by Mr. EDDIE, the dresser at the hospital: the subjoined remarks are by Mr. KEY.—EDITOR.

years old, since which time they gradually increased, and, when admitted, she was labouring under some degree of constitutional excitement; her pulse was quick, tongue furred, and skin dry. On sounding her, soon after her admission, a calculus could be easily felt, which appeared rather large. The meatus urinarius was ordered to be gradually dilated by means of bougies, with the intention of making a more accurate examination of the stone.

After the meatus had been sufficiently dilated, and the general health improved by strict attention to her bowels and moderate diet, a small pair of forceps was introduced into the bladder, and, by means of this, the calculus was found to be so large that it would be almost impossible to remove it by the urethra. Soon after this, an attempt was made to crush the stone; but this was unsuccessful, from the difficulty of keeping the bladder distended.

Mr. Key now determined upon the high operation; but it was not performed, on account of her having a slight attack of fever, until the 25th September, at which time she was much better in health than she had been since her admission, although her pulse was still quick, and the tongue rather furred; her bowels, however, were regular. The operation was performed in the following manner:

The little patient was placed on her back on the operating table, with her shoulders raised, and her legs hanging over the end, with the feet resting on a chair. About six ounces of tepid water were injected into the bladder, to prevent the return of which, the fore finger of an assistant was passed into the vagina, in order to compress the meatus against the symphysis pubis. An incision was then made in the median line, to the extent of two inches, immediately above the symphysis pubis: this was continued carefully between the fibres of the pyramidales muscles, until a quantity of adipose membrane covering the anterior surface of the bladder was visible. A sharp-pointed bistoury was now plunged into the bladder, and the operator, having introduced the fore finger of the left hand into the opening thus made, was enabled in some measure to raise the bladder, and thus extend the incision downwards. The situation of the stone having been ascertained by the finger in the bladder, the forceps were introduced, and the stone extracted without much difficulty. After the operation the patient was placed on her back in bed, with her knees raised by a pillow; a piece of dry lint was applied over the wound, the edges of which were approximated in some measure by means of adhesive plaster.

Two P.M. (two hours after the operation.) The patient whose pulse was 130, complained of a sharp pain in the bladder. As she had not passed any urine, the catheter was introduced, and about four ounces of thick bloody urine came away.—Sumat Mist. Amygdalæ cum Nitro quartis horis.

Ten P.M. Was much relieved by the introduction of the catheter. Has been quite easy, and slept a little in the evening. The urine continued to flow by the urethra involuntarily until about an hour ago, when it stopped, and has now begun to trickle through the wound: to prevent this, a metallic catheter was introduced into the bladder, and a few drachms of very thick bloody urine came away; and this instrument being soon choked by coagulum, was removed, and an elastic one, with larger apertures, was introduced in its stead: through this the urine trickled guttatim, at first bloody, but afterwards clear. This at first created much uneasiness, which was soon relieved by the administration of two drachms of Syr. Papav., and she enjoyed a good night.

September 26th. Eight A.M. The catheter creates no uneasiness, and a considerable quantity of water has passed by it in the night; pulse 130; tongue furred, but not dry; and skin moist. On pressing the abdomen, there is no general tenderness, but only some pain in the wound; she has, however, had some little vomiting.—Ordered to leave off her mixture, and to take Mist. Effervesc. quartis horis.

27th, A.M. Appears to be going on well: the water has continued to flow by the catheter, and she has slept several hours during the night; pulse 120; skin cool and moist. As her bowels have not been moved since the operation, the following mixture was prescribed: ℞. Mist. Magnesiæ cum Magnes. Sulph. f℥i. sumat secundis horis ad alvi solutionem.

28th, A.M. The medicine ordered yesterday operated three times during the day, and once in the night. Part of the dressing having become loose, was removed, and the rest which was adherent being very hard, was covered with a bread poultice. The catheter was removed in the middle of the day, to see whether the urine would pass by the urethra; but, as it had begun in the evening to make its appearance in the wound, the instrument was re-introduced. The patient has slept pretty well during the night; her pulse is 120; tongue furred, but moist; skin hot. She complains of thirst, has no appetite, and cannot be prevailed on to take anything. The wound is now left open, to heal by granulations, and some simple dressing is applied to it. She has resumed the effervescing mixture.

29th. She was rather better this morning: pulse 120, skin cooler and moist, tongue cleaner; but towards evening was not so well. Pulse 130; tongue dry and coated; complains of thirst, and can take no food; the effervescing mixture is immediately vomited, and barley-water is the only thing she can take. She complains of no pain or tenderness of the abdomen, excepting in the wound; the urine continues to pass by the catheter.

30th. The eruption of scarlatina has made its appearance, and the patient is rather better than last night: tongue moist, furred in the centre, and red round the edges; pulse 125.*

October 1st. Going on well: tongue very red, moist and not furred; rash increased; pulse 128; complains of no pain; skin hot, and rather dry. Bowels have been twice moved; the urine passes freely by the catheter. She has hitherto lain on her back, which has become irritated by the urine; she is therefore ordered to lie on her side.—℞. Hydr. Subm. gr. i.; Pulv. Antimon. gr. ij.; Pulv. Tragacanth. comp. gr. iij. h. s. s.

2d. Has passed a very restless night: the powder was rejected soon after it was taken; the pulse still quick, but weak and compressible; tongue moist, skin cool, and bowels open. There is very little suppuration from the wound, which has a sloughy appearance; the rash is dying away.—Ordered beef-tea; a piece of lint, wet with Lotio Chlor. Sodæ, to be kept in the wound under the simple dressing. Adde sing. dos. Misturæ Tinct. Calumbæ, ʒss.

3d. Much better. The beef-tea and medicine have staid on her stomach, and she took last night, in addition, a tea-cupful of sago with a little wine in it; and she passed a good night. Pulse 125, and somewhat stronger than yesterday.—Pergat.

4th. Much the same as yesterday; passed a good night; bowels are open; and the wound begins to secrete healthy pus, some of which passes by the catheter.—Pergat.

5th. Much the same.—℞. Decoct. Cinchonæ, ʒvijss.; Acidi Sulph. dil. m. vi.; Tinct. Aurant. ʒss. M. ut fiat haust. sextis horis sumendus.

12th. Has gone on pretty well since last report; sleeps well, appetite good, tongue moist, bowels open: her back, which has been much irritated, is somewhat better. The wound is closing by granulations, but the water has passed by it for several days. The catheter, becoming choked with mucus, was ordered to be withdrawn. The patient, having had some cough since yesterday, was ordered to omit the mixture, and take the Mist. Mucil.

14th. The mucilaginous mixture makes her sick, and her skin is somewhat dry.—℞. Potassæ Nit. gr. v. cum Tinct. Hyoscyami, m. v., et Mist. Salin. ter die.

* The treatment of the scarlatina was under Dr. Cholmeley.

18th. Going on pretty well; pulse 110; appetite good, and she begins to take a little meat. The bowels are open, but the tongue is rather furred; the skin dry and harsh. The wound produces healthy granulations, though the urine has continued to flow through it almost entirely since the catheter was withdrawn.—Thermæ. Pergat in aliis.

21st. Continues to improve; eats and sleeps well; pulse 100, skin soft and moist, tongue clean, bowels open. The wound is closing, and she has passed a little urine by the urethra, though the greater part still flows through the wound.—Ordered to sit up a short time every day. Pergat.

25th. Going on well. The wound is closing; she is not able to sit up long, but the erect position causes the water to pass more readily by the urethra: still, however, some passes by the wound.—Pergat.

29th. Continues to improve; the urine passes almost wholly by the natural passage, though involuntarily, and there is some catarrhal affection of the bladder. She is now able to sit up the greater part of the afternoon.—Sumat Balsami Copaibæ, m. v. ter die.

November 8th. Improving fast in all respects. The wound is nearly healed, except a very small aperture, from which a minute quantity of pus is discharged, but no urine. She is now able to walk a little in the ward. The catarrhal affection of the bladder is relieved, but there is still some incontinence of urine.—Sumat Decoct. Uva Ursi, ℥i. ter die.

After this she improved rapidly, and left off the medicine in a week, the incontinence of urine being relieved.

November 26th. The wound had healed, and she left the hospital apparently quite well in every respect, being able to retain her urine as before the operation, and not requiring to void it oftener than three or four times in the course of the day.

My first intention, when this little girl came under my care, was, if possible, to remove the calculus by dilating the canal; this, however, I determined to do with some limitation, as I had witnessed, in more than one case, incontinence of urine follow the operation of dilatation, when the calculus was large in proportion to the size and age of the patient. The first examination at once convinced me that it could not be removed entire without risking the consequence of that worst of evils to a female, incontinence of urine; and this led me to consider the possibility of breaking it in the bladder, and removing it piecemeal. But the difficulty of preventing the escape of the water during the operation, and the extreme sensibility of the surface of the bladder after a trial, rendered this operation in my view objectionable. The lateral operation in the female is one of great simplicity, and requires but little skill or anatomical knowledge for its performance; but it so often leaves the patient with an imperfect control over the sphincter vesicæ, that I judged it more prudent even to incur the risk of a somewhat hazardous operation, than to doom her probably to the condition of being loathsome to herself, and a nuisance in society.

Desirous therefore of making everything connected with the operation subservient to the object of preserving the power over the neck of the bladder, after some consideration I resolved upon performing the operation above the pubis, and thus leaving the neck of the bladder entire. I had never performed the operation, nor had I ever seen it performed by other surgeons; and the results of the few cases in this country that had come to my knowledge rendered me cautious in the steps to be pursued. There are two sources of danger in the operation; and upon the avoiding these will mainly depend its success. These are the risk of wounding the peritoneum in the operation, and of urinary infiltration after it.

The danger of wounding the peritoneum in opening the bladder, will not, I think, be very great, if the condition of that viscus be such as to enable the operator to distend it well with water, and thus to carry the peritoneal investment, as the bladder rises above the pelvis, away from the point of incision. In the young subject the fundus of the bladder receives so small a covering from the peritoneum, that very moderate distention will carry the peritoneum beyond the reach of a careful operator; in the adult, a much larger quantity of fluid must be injected to effect this purpose; and in cases when the state of the bladder will not allow of very complete distention, I should much question the propriety of attempting the operation. The first incision through the integuments and linea alba requires merely common dissection, and should be as free as the size of the patient will allow; it should not, however, at first be carried to the full extent, as the bladder, when laid bare, will point out to the operator whether he has made his incision as high as it can be carried without endangering the peritoneum. At first, therefore, the linea alba should be just sufficiently divided to enable the finger to feel the distended bladder.

The extent of space between the tendon and the bladder will enable the operator to judge pretty clearly what part of the bladder he has exposed; whether he is in contact with the fundus where the peritoneum is reflected from it, or with the free muscular coat nearer the neck of the viscus. This will be understood when it is remembered that the anterior part of a distended bladder is not parallel to the linea alba, but that it forms an angle with it; the apex being at the upper part where the peritoneum covers it, the hypothenuse at the lower part towards the neck; and thus, the greater the space between the tendon and the bladder, the greater will be the assurance that the peritoneum is out of danger. The place for puncture being selected, a sharp-pointed bistoury is plunged into the bladder, and carried downward toward the pubes, so as to make an opening sufficient to admit the finger. This plunge of the knife, and the introduction of the finger, must be the work of an instant, as the water immediately escapes, and the opening in the bladder recedes from that in the integument. The bladder, thus hooked upon the finger, can then be more freely opened by carrying the bistoury down toward the cervix. The site and form of the stone being ascertained, the remaining part of the operation consists in seizing it with the forceps, and extracting it. This step is not so readily effected as might at first view be supposed, owing to the firm contraction of the pyramidales muscles. In the present case the resistance was not great; but I can readily imagine a case in which these muscles, in the adult, would embarrass the operation, and a partial division of one or both might be required to liberate the stone.

It will be observed, that a catheter was not introduced immediately after the operation. It appeared probable that the coagula in the bladder would choke it up, and prevent the water flowing through it; and the presence of a catheter might add to the risk of inflammation. I therefore directed Mr. Eddie to introduce the catheter as soon as she began to experience any desire to empty the bladder, which I apprehended would soon be felt, from the oozing of blood that would continue to take place into its cavity. It is desirable to keep the bladder as empty, and thereby as quiet as possible, to enable it to adhere to the surrounding cellular membrane, and prevent the extravasation of urine. Nature herself, I suspect, guards effectually against this accident, by letting the water drain out of the bladder as far as it is secreted, instead of it collecting in its cavity, and distending its coats. A large opening also lessens the chance of infiltration; and I imagine this disastrous event to be much less likely to follow an operation for lithotomy than for puncturing the bladder for retention of urine, in which the opening into the bladder, as also in the integuments, is small.

It is doubtful whether any good is derived from keeping a catheter in the bladder during the cure, with a view of draining the water off, and preventing it finding its way through the wound. In some respects it is attended with disadvantage; for, by constantly keeping the bladder empty, it is kept in a permanent state of contraction, which, though at first an advantage, by preventing extravasation, becomes afterwards an impediment to the healing of the wound. For, if the water could not find its way by the meatus, in the contracted state of the bladder, it must be constantly dribbling away by the wound; but, as soon as the catheter is withdrawn, the bladder becomes expanded by the collection of water, which only forces its way through the wound when the viscus contracts: and it was observed that the wound healed faster, and the bladder held more urine, after the catheter was withdrawn altogether.

The calculus measured in its longest circumference four inches and four tenths, and in its shortest circumference three inches and two tenths. For so young a child this was a large stone.

From the favourable result of this case, under certainly unpromising circumstances, I should feel disposed to practise the operation where circumstances prevented the meatus being dilated: its advantage over the lateral operation in the female is, in my mind, decided; and, in the case of a large stone in the male subject, I do not see any impediment to its success.

